# A study of calibrating seismic design response spectrum based on high-intensity ground motion records

**DOI:** 10.1038/s41598-026-58966-1

**Published:** 2026-06-22

**Authors:** Qian Liang, Bai Yunpeng, Xie Quancai, Zhang Xiaonan, Li Jilong, Wang Yuhuan

**Affiliations:** 1 Key Laboratory of Earthquake Engineering and Engineering Vibration, Institute of Engineering Mechanics, Earthquake Administration, Harbin, 150080 Heilongjiang China; 2 Key Laboratory of Earthquake Disaster Mitigation, Ministry of Emergency Management, Harbin, 150080 Heilongjiang China; 3 Tianjin Earthquake Agency, Tianjin, 300201 China; 4 School of Earthquake Engineering and Building Safety, University of Emergency Management, Sanhe, 065200 China; 5 Key Laboratory of Building Collapse Mechanism and Disaster Prevention, China Earthquake Administration, Sanhe, 065200 China

**Keywords:** Calibration of the design response spectrum, Chinese seismic intensity, Adaptive guided differential evolution algorithm, Spectral parameters, Site conditions, Engineering, Natural hazards, Solid Earth sciences

## Abstract

The seismic design response spectrum is a vital parameter for determining the potential seismic load of the engineering structure. Differential evolution algorithm (DE) with a novel hybrid mutation operator is utilized to calibrate the spectral parameters in order to enhance iteration efficiency. This study calibrates the seismic design response spectrum for China based on the Chinese seismic intensity scale and compares the results with the Code for Seismic Design of Buildings (CSDB2010).The calibration spectra are based on strong ground motion records with destructive power exceeding the Chinese seismic intensity 7 and above. The characteristics of the calibration spectral parameters are analyzed and subsequently compared with the design spectra of the Code for Seismic Design of Buildings (CSDB2010). It is found that: Increasing the number of iterations can enhance the fitting goodness of DE between the calibration spectrum and the record response spectrum. The average site characteristic period (*T*_g_) for rock and hard soil site conditions (Class I and II) is greater than the *T*_g_ specified in CSDB2010. The average *T*_g_ for intensity 10 + is greater than the average *T*_g_ for intensity 7, 8, and 9. *T*_g_ increases with the seismic intensity. The average spectra platform value (*β*_max_) from this study gradually approaches the *β*_max_ in CSDB2010 as the intensity increases. The average attenuation index (*γ*) at different intensities of this study is greater than *γ* in CSDB2010.

## Introduction

The seismic design response spectrum serves as a fundamental parameter in seismic engineering design for determining potential seismic loads on structures. Newmark and Hall^1^ first proposed three parameters—peak ground acceleration (PGA), peak ground velocity (PGV) and peak ground displacement (PGD)—to smooth design seismic response spectra, forming the well-known three-parameter calibration method. This method has been widely adopted in international building codes such as EC8^2^ and ASCE2002^3^. Hayashi et al.^4^ selected numerous accelerogram records under different soil conditions in Japan and classified response spectra into three groups, representing one of the earliest studies on the influence of site conditions on response spectral characteristics. Malhotra^5,6^ introduced the concept of the central period of ground motion as a critical indicator for smoothing design response spectra, enabling smoothed spectra to exhibit reasonable asymptotic characteristics in both short- and long-period ranges. Chopra and Choudhury^7^ investigated the influence of local geological conditions on acceleration response spectra using observational data from 32 sites in Gujarat, India. Bommer et al.^8^ formulated seismic design response spectra for nuclear power plant engineering in the United Kingdom. Calvi et al.^9^ established new design spectra based on Italian ground motion records collected from 1972 to 2017, and further revealed the combined effects of local soil conditions, earthquake magnitude and source-to-site distance on empirical design response spectra^10,11,41,42^.

The differential evolution (DE) algorithm is a heuristic optimization approach designed to search for global optimal solutions. It performs random searching within a given parameter range and is suitable for the direct fitting of multi-parameter functional models^12,13^. This algorithm is well-suited for fitting problems where the functional form is explicitly known but the characteristic parameters remain undetermined. Shi et al.^14^ verified that the DE algorithm outperforms the simulated annealing algorithm in the calibration of seismic design spectra.

**Fig. 1:**
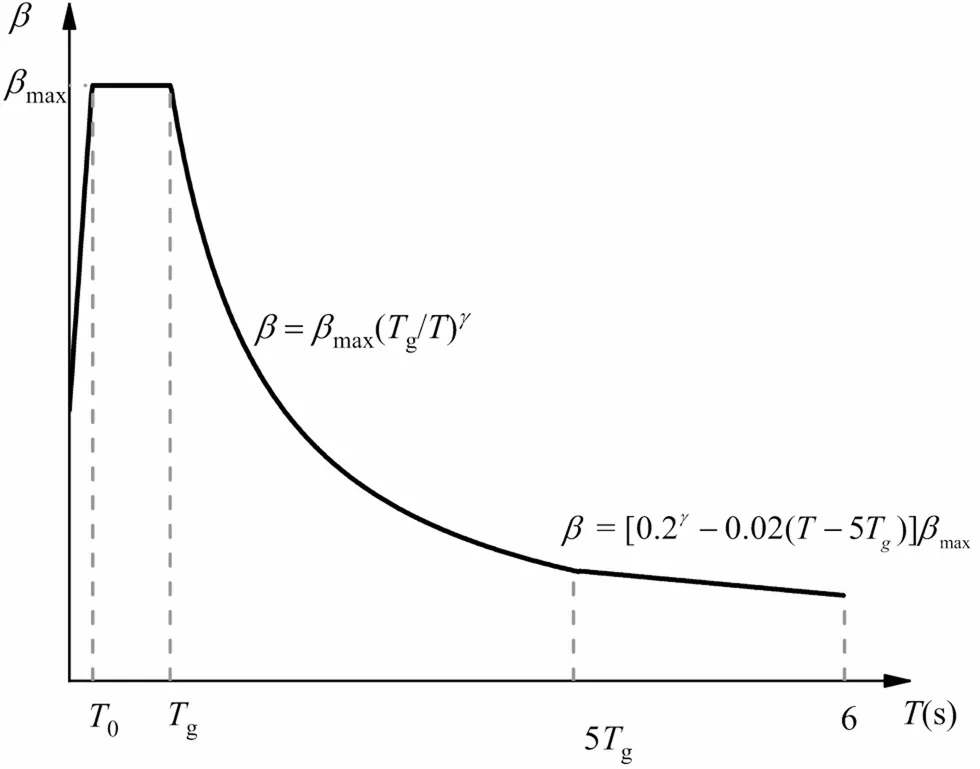
The curved shape of the normalized response spectrum.

For the purpose of analysis and comparison, this study transforms the response spectra into standardized amplification factor spectra, which is the ratio of response spectra values to peak ground acceleration. The transformed design spectra are as follows^15^:

1 $$\beta \left( T \right){\text{ = }}\left\{ \begin{gathered} 1 + \frac{{\beta _{{\max }} - 1}}{{T_{0} }}T{\text{ 0}} \le T < T_{0} \hfill \\ \beta _{{\max }} {\text{ }}T_{0} \le T < T_{g} \hfill \\ \beta _{{\max }} (\frac{{T_{g} }}{T})^{\gamma } {\text{ }}T_{g} \le T < 5T_{g} \hfill \\ [0.2^{\gamma } - 0.02(T - 5T_{g} )]\beta _{{\max }} {\text{ 5}}T_{g} \le T < 6 \hfill \\ \end{gathered} \right.$$

where *β*(*T*) represents the amplification factor spectral value, *T* is the spectral period, *T*_0_ is the first corner period, βmax is the spectra platform value, *T*_g_ is the site characteristic period, and γ is the attenuation index. Figure 1 shows the shape of *β*(*T*). The Code for Seismic Design of Buildings (CSDB2010) only divide site characteristic periods into different fixed intervals based on site categories, without considering the potential variation of spectral parameters under different seismic intensity levels, which may lead to deviations in seismic load calculation for structures in high-intensity seismic regions. Therefore, this paper collects a large number of actual strong-motion observation records from regions that experienced seismic intensity exceeding 7 in China, and adopts the adaptive guided differential evolution algorithm to calibrate the four parameters of the standardized amplification factor design spectrum for different site conditions and seismic intensity levels. The statistical distribution characteristics of the calibrated parameters are analyzed, and the results are compared.

In the standard differential evolution (DE) algorithm, the two parameters *F* (scaling factor) and *CR* (crossover rate) are fixed values, which reduces the optimization efficiency of the algorithm. To address this limitation, many scholars have developed improved DE strategies with adaptive parameter adjustment to enhance algorithm performance. Liu et al.^16^ proposed the fuzzy adaptive DE (FADE) algorithm, which uses the adaptive adjustment mechanism of a fuzzy logic controller to control parameters. Santana-Quintero and Coello^17^ proposed the ε-MyDE algorithm, which was the first to incorporate the concept of ε-dominance to achieve a uniform distribution of retained solutions.

On this basis, a series of improved DE variants have been proposed^18^; these include Trigonometric Differential Evolution (TDE)^19^, Opposition Based Differential Evolution (OBDE)^20^, Self-adapting Parameter Setting in Differential Evolution^21^, Self-adaptive Hybrid Differential Evolution (SaDE)^22^, and others^23–30^.

In the response spectra calibration process of this study, the root mean square error is used as the objective function of DE, so that the calibrated response spectra can better fit the recorded spectra. However, the shape of response spectra is related to site conditions and ground motion characteristics. This study still uses the root mean square error as the objective function of DE during response spectra calibration, which allows the calibrated response spectra to better fit the recorded spectra.

In this study, high-quality strong-motion records from near-fault stations are selected to calculate the Chinese seismic intensity. Records with high Chinese seismic intensities (7, 8, 9, 10, and above) are selected to calibrate the record response spectra using the DE algorithm. The fitting effects are then compared with records’ response spectra. The distribution of calibration spectra parameters is analyzed. The variation trend of *β*_max_, *T*_g,_ and *γ* parameters of the calibrated design spectra with respect to the site average shear wave velocity of the top 30 m (referred to as *V*_*s30*_) is analyzed. This study provides a reference for the improvement of seismic design response spectra parameters.

## Methodology of Chinese seismic intensity

In order to study the relationship between parameters of strong ground motions and instrument seismic intensity, Trifunac et al.^31^ calculated the correlation between the intensities of different stations with strong ground motions and PGA and PGV. United States Geological Survey (USGS) established the ShakeMap system to release disaster information, in which the calculation method of the seismic intensity of the system’s instruments mainly referred to PGA and PGV^32,33^. Similar to the calculation method of seismic intensity used in the ShakeMap system of USGS, the PGA and PGV are also used as the physical scale of seismic intensity in China. The main reference for instrument seismic intensity in the ShakeMap system is the modified Mercalli intensity scale. The Chinese Seismic Intensity Scale (GB/T 17742 − 2020)^34^ was compiled on the basis of revising the Mercalli intensity scale and combining the actual situation of earthquake damage in China.

The Chinese Seismic Intensity Scale (GB/T 17742 − 2020) provides detailed instructions on the calculation method for instrumental seismic intensity. The steps are as follows:

(1) Baseline correction and Digital filtering. The method for baseline correction of seismic motion records involves subtracting the arithmetic average of the pre-seismic background noise from the seismic record. In general, the duration of the background noise is selected as 10 s. Digital filtering. Acceleration and velocity records of the east-west (EW), north-south (NS), and vertical (UD) directions components all require bandpass filtering within the range of 0.1 to 10 Hz. The filter passband ripple should not exceed 0.5 dB, and the filter out-of-band attenuation should be greater than 12 dB/octave.

(2) Records synthesis. The following formulas are used to calculate the three-component synthesized acceleration and velocity time histories:

2 $$a(t_{i} ) = \sqrt {a^{2} (t_{i} )_{{{\mathrm{EW}}}} + a^{2} (t_{i} )_{{{\mathrm{NS}}}} + a^{2} (t_{i} )_{{{\mathrm{UD}}}} }$$

3 $${\mathrm{v}}(t_{i} ) = \sqrt {{\mathrm{v}}^{2} (t_{i} )_{{{\mathrm{EW}}}} + v^{2} (t_{i} )_{{{\mathrm{NS}}}} + v^{2} (t_{i} )_{{{\mathrm{UD}}}} }$$

In which, *a*(*t*_*i*_)_EW_, *a*(*t*_*i*_)_NS_, and *a*(*t*_*i*_)_UD_ represent acceleration values in the east-west, north-south, and vertical direction components at time *t*_*i*_, respectively. *v*(*t*_*i*_)_EW_, *v*(*t*_*i*_)_NS_, and *v*(*t*_*i*_)_UD_ represent the filtered velocity values in the east-west, north-south, and vertical directions at time *t*_*i*_.

(3) Calculate the three-component synthesized seismic parameters PGA and PGV.

(4) Calculate the Chinese instrumental seismic intensity. The following formulas are used to calculate *I*_A_ and *I*_V_:

4 $$I_{{\mathrm{A}}} = 3.17\log _{{10}} (PGA) + 6.59$$

5 $$I_{{\mathrm{V}}} = 3.00\log _{{10}} (PGV) + 9.77$$

The instrumental seismic intensity (abbreviated as *I*_china_) is calculated using the formula (6), and the result can be two significant digits after the decimal point. If *I*_china_ is less than 1.0, it should be rounded up to 1.0. If *I*_china_ is greater than 12.0, *Ichina* equals 12.0.

6 $$I_{{{\mathrm{china}}}} = \left\{ {\begin{array}{*{20}c} {I_{V} } \\ {(I_{A} + I_{V} )/2} \\ \end{array} } \right.\begin{array}{*{20}c} {{\text{ }}I_{A} \ge 6.0{\text{ and }}I_{V} \ge 6.0} \\ {{\text{ }}I_{A} < 6.0{\text{ or }}I_{V} < 6.0} \\ \end{array}$$

### Dataset of strong-motion

In this study, a total of 452 strong motion records were selected from six major earthquake events. Detailed information about the earthquakes is listed in Table 1. The records were sourced from the Pacific Earthquake Engineering Research Center (PEER), the Japanese KiK‑net and K‑net networks, and the Institute of Engineering Mechanics, China Earthquake Administration (IEM). The moment magnitude (Mw) and hypocentral depth of each event were obtained from the official websites of the Japanese F‑net network and the U.S. Geological Survey (USGS) to ensure data authenticity and reliability. The Mw of the selected events ranges from 6.7 to 9.1. The hypocentral depth ranges from 8 to 24 km. The selected events are well balanced in terms of basic information, and the number of records per event is relatively consistent, which helps reduce potential biases caused by uneven data distribution.

**Table 1:** Details of the earthquakes in the database.

Event name	Time	Depth(km)	M_w_	record number
Northridge	1994/1/17	18	6.7	54
Kobe	1995/1/16	10	6.9	24
Chichi	1999/9/20	11	7.6	104
Wenchuan	2008/5/12	14	7.9	22
Tohoku	2011/3/11	24	9.1	184
Turkey	2023/2/6	8.6	7.7	64

The following criteria were applied to select the 452 records: (1) Fault distance: records were taken from stations with a rupture distance of less than 150 km to focus on near-field strong motions. (2) Waveform completeness: only three‑component records with no significant gaps or missing data were included. (3) Signal-to-noise ratio (SNR): each record is required to have SNR > 3. (4) Shear-wave velocity information: the time-averaged shear-wave velocity in the upper 30 m (*V*_s30_) at the station must be available, either from direct measurement or through reliable empirical estimates. (5) Chinese seismic intensity: only records from stations with Chinese seismic intensity of VII or above were retained. Before analysis, all raw acceleration records were processed as follows. Baseline correction was performed by subtracting the mean value of the first 10 s of the acceleration record from the original data to remove baseline drift. Subsequently, a non-causal fourth-order Butterworth band-pass filter was applied. The low-cut frequency was set between 0.02 and 0.1 Hz depending on the quality of each record, while the high‑cut frequency was fixed at 30 Hz.

**Fig. 2:**
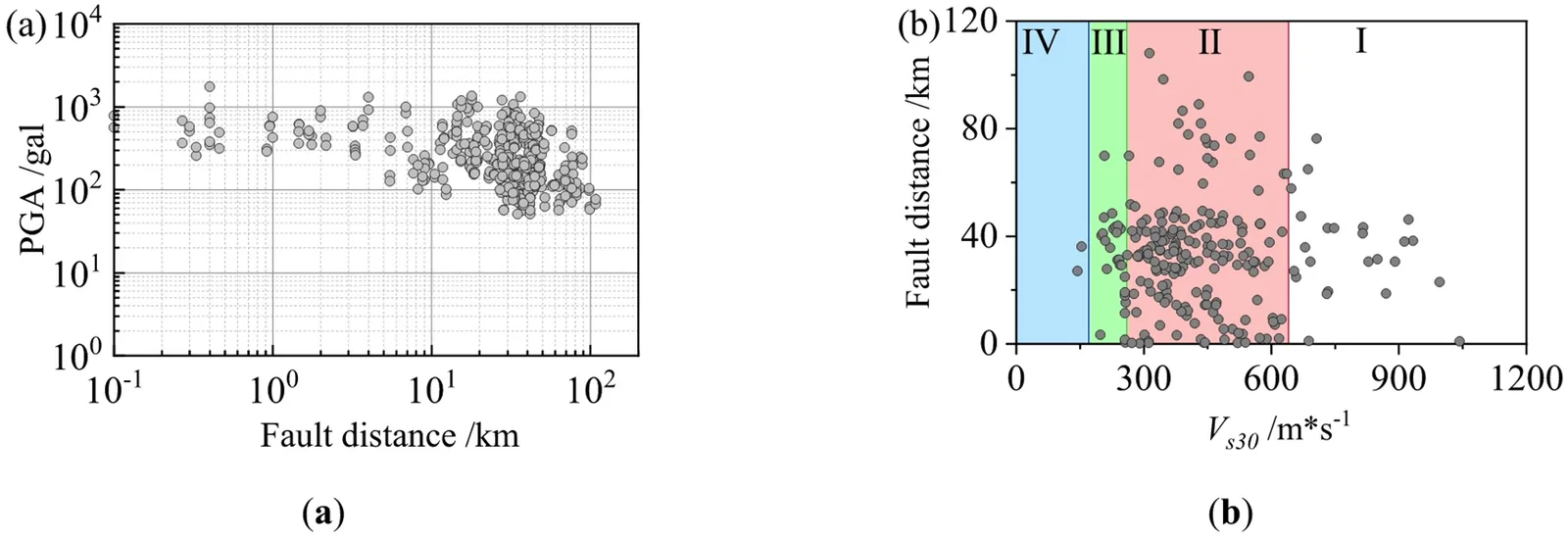
(**a**) Distribution of records with respect to PGA (gal) and fault distance (km); (**b**) Distribution of records with respect to fault distance (km) and *V*_s30_ (m/s).

Figure 2(a) shows most of the records are above 100 gal, while a few are above 1000 gal. fault distances of most records are between 20 and 100 km, including 44 records with fault distance less than 5 km. The stations are classified according to the conversion method between site average shear wave velocity of the top 30 m (*V*_s30_) and Chinese site classification proposed by Xie et al.^35^. It can be seen from Fig. 2(b) that *V*_s30_ of most stations is between 300 and 600 m/s, *V*_s30_ of 9 stations is above 760 m/s, and *V*_s30_ of few stations was less than 150 m/s. Most stations belong to site classes II and III, while a few stations belong to site classes I. The selected ground motions for this study are shown in Table 1. The *M*_w_ range is 6.7–9.1 and the depth range is between 10 and 20 km.

**Fig. 3:**
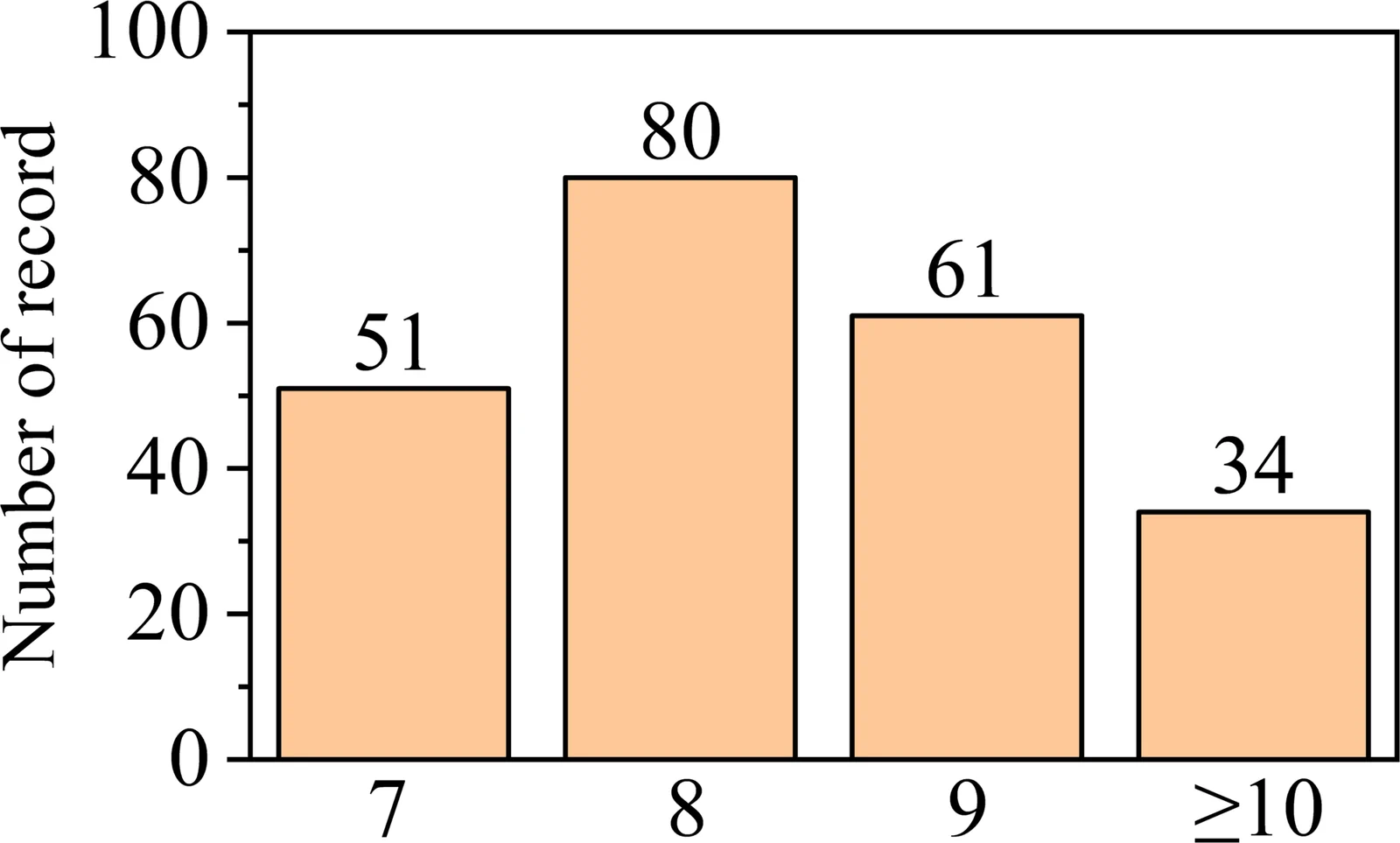
Number of records at different Chinese seismic intensity levels.

Figure 3. shows the distribution of the number of records of intensity 7, intensity 8, intensity 9, intensity 10+. The records of strong ground motions selected in this study are mostly intensity 8 or 9. The number of intensity 7 records is 51. The number of intensity 8 records is 80. The number of intensity 9 records is 61. The number of intensity ≥ 10 (referred to as 10+) records is 34.

### Differential evolution algorithm (DE)

The general steps of the differential evolution algorithm are as follows: (1) Initialization; (2) Mutation operation; (3) Crossover operation; (4) Selection operation. The initial parameters are population size NP (number of candidate solutions) and max evolution generations Gmax (max number of iterations). Storn and Price^13^ recommended that NP usually take a value between 5D and 10D (D is the dimension of solutions). Gmperle et al.^17^ summarized the test results and concluded that the performance of DE algorithm depended heavily on the setting of control parameters, and the control parameters were set as follows: the number of populations NP and the ideal interval was 3D-8D. Ronkkonen et al.^27^ believed that the ideal interval for the population number NP was 2D~40D. As computer performance improves, more data and calculations become possible. Based on the suggestion of Ronkkonen et al.^25,27^ D is considered the proper population number for this study.

The target vectors of *g*th generation (*X*_*g*_), are as follows:

7 $$X_{g} = \left[ {\begin{array}{*{20}c} {X_{{1,g}} } \\ {X_{{2,g}} } \\ \vdots \\ {X_{{NP,g}} } \\ \end{array} } \right] = \left[ {\begin{array}{*{20}c} {x_{{1,g}}^{1} } & {x_{{1,g}}^{2} } & \cdots & {x_{{1,g}}^{D} } \\ {x_{{2,g}}^{1} } & {x_{{2,g}}^{2} } & \cdots & {x_{{2,g}}^{D} } \\ \vdots & \vdots & \vdots & \vdots \\ {x_{{NP,g}}^{1} } & {x_{{NP,g}}^{2} } & \cdots & {x_{{NP,g}}^{D} } \\ \end{array} } \right]$$

In which, g represents the evolution generations, g = 1, 2,…, *G*_max_. D represents dimension of solutions. The dimension of solutions in this study is 4, which are respectively the first corner period (*T*_0_), the spectra platform value (βmax), the site characteristic period (*T*_g_), and the attenuation index (*γ*). The iteration steps of the DE are as follows:

(1) Initialization:

This study randomly initializes initial generation (*g* = 0) target vectors in the search space. The initialization can be defined as:

8 $$X_{0} = X_{{\min }} + rand(0,1)*(X_{{\max }} - X_{{\min }} )$$

In which, Xmin and Xmax are the lower bound and upper bound in each dimension. rand (0, 1) is a random number between 0 and 1.

9 $$X_{g} = \left[ {\begin{array}{*{20}c} {X_{{1,0}} } \\ {X_{{2,0}} } \\ \vdots \\ {X_{{NP,0}} } \\ \end{array} } \right] = \left[ {\begin{array}{*{20}c} {x_{{1,0}}^{1} } & {x_{{1,0}}^{2} } & \cdots & {x_{{1,0}}^{D} } \\ {x_{{2,0}}^{1} } & {x_{{2,0}}^{2} } & \cdots & {x_{{2,0}}^{D} } \\ \vdots & \vdots & \vdots & \vdots \\ {x_{{NP,0}}^{1} } & {x_{{NP,0}}^{2} } & \cdots & {x_{{NP,0}}^{D} } \\ \end{array} } \right]$$

(2) Mutation.

Yi et al.^36^ proposed a self-adapting mutation in DE, named the novel hybrid mutation operator. The proposed algorithm can balance exploration and exploitation well. The mutation vectors(*V*_g_) of gth generation, can be follows:

10 $$V_{g} = \left[ {\begin{array}{*{20}c} {V_{{1,g}} } \\ {V_{{2,g}} } \\ \vdots \\ {V_{{NP,g}} } \\ \end{array} } \right] = \left[ {\begin{array}{*{20}c} {v_{{1,g}}^{1} } & {v_{{1,g}}^{2} } & \cdots & {v_{{1,g}}^{D} } \\ {v_{{2,g}}^{1} } & {v_{{2,g}}^{2} } & \cdots & {v_{{2,g}}^{D} } \\ \vdots & \vdots & \vdots & \vdots \\ {v_{{NP,g}}^{1} } & {v_{{NP,g}}^{2} } & \cdots & {v_{{NP,g}}^{D} } \\ \end{array} } \right]$$

A novel hybrid mutation operator is used. For each individual in the population, this study selects five random mutually different indexes (*r*1, *r*2, *r*3, *r*4, *r*5). A mutant vector is Generated by combining the information from the parent individuals using the mutation formula:

11 $$V_{{i,g}} = \left\{ {\begin{array}{*{20}l} {X_{{r3,g}} + F_{{i,g}} * (X_{{r4,g}} - X_{{r5,g}} ){\text{ when }}f{\mathrm{(}}X_{{i,g}} {\text{) > }}f{\mathrm{(}}X_{{r1,g}} {\text{) and }}f{\mathrm{(}}X_{{i,g}} {\text{) > }}f{\mathrm{(}}X_{{r2,g}} {\mathrm{)}}} \hfill \\ {X_{{i,g}} + F_{{i,g}} * (X_{{best,g}} - X_{{r5,g}} ) + F_{{i,g}} * (X_{{r4,g}} - X_{{r5,g}} ){\text{ others}}} \hfill \\ \end{array} } \right.$$

In which, *Fi*,* g* represents scaling factor of *i*th individual of *g*th generation, *f*(*Xi*,* g*) is objective function value of *X*_i, g_. For a randomly selected individual *X*_rl_ and *X*_r2_ the probability that its function value is worse than that of the individual *X*_i_ is 0.5, and the same applies for *X*_r2_.Because *X*_r1_ and *X*_r2_ are separately selected, the selection of *X*_r1_ and *X*_r2_ are independent random events; therefore, the possibility of *f*(*X*_*i, g*_) > *f*(*X*_*r1,g*_) and *f*(*X*_*i, g*_) > *f*(X_r1,g_) is 0.25.

In practical applications, if F is set as a constant and F is too large, the convergence speed of the algorithm will be slowed down and the accuracy of the global optimal solution obtained will be reduced. If F (scaling factor) is too small, the diversity of the population will be reduced and prematurity will occur. Therefore, parameter F can be set to a value that changes with the number of iterations. With the increase of the number of iterations, F decreases, which can preserve excellent population information and avoid destroying the optimal solution. Self-adaptive parameter control strategy is used in F.According to different objective functions, Liu et al. proposed different methods to definite the value of F. The formula is as follows:

12 $$F_{{i,g + 1}} = \left\{ {\begin{array}{*{20}l} {F_{l} {\text{ + }}rand{\mathrm{(0,1)}} * (F_{u} {\text{ - }}F_{l} {\text{) }}f{\mathrm{(}}U_{{i,g}} {\text{) > }}f{\mathrm{(}}X_{{i,g}} {\mathrm{)}}} \hfill \\ {F_{{i,g}} {\text{ }}f{\mathrm{(}}U_{{i,g}} {\mathrm{)}} \le f{\mathrm{(}}X_{{i,g}} {\mathrm{)}}} \hfill \\ \end{array} } \right.$$

In which, *F*_*l*_ represents the lower scaling factor, Fl = 0.1. Fu represents the upper scaling factor, *F*_*l*_=0.9.

(3) Crossover:

After mutation, a crossover operator is applied to enhance the diversity of the population. The *g*th generation mutation vectors (*U*_*g*_), can be follows:

13 $$U_{g} = \left[ {\begin{array}{*{20}c} {U_{{1,g}} } \\ {U_{{2,g}} } \\ \vdots \\ {U_{{NP,g}} } \\ \end{array} } \right] = \left[ {\begin{array}{*{20}c} {u_{{1,g}}^{1} } & {u_{{1,g}}^{2} } & \cdots & {u_{{1,g}}^{D} } \\ {u_{{2,g}}^{1} } & {u_{{2,g}}^{2} } & \cdots & {u_{{2,g}}^{D} } \\ \vdots & \vdots & \vdots & \vdots \\ {u_{{NP,g}}^{1} } & {u_{{NP,g}}^{2} } & \cdots & {u_{{NP,g}}^{D} } \\ \end{array} } \right]$$

A binomial crossover is applied to combine the mutant vector with the target vector to create a trial vector. mutation vectors(*U*_*g*_) are obtained using the Equation:

14 $$u_{{_{{i,g}} }}^{j} = \left\{ {\begin{array}{*{20}l} {v_{{_{{i,g}} }}^{j} {\text{ if }}rand{\text{(0,1) }} \le CR} \hfill \\ {x_{{_{{i,g}} }}^{j} {\text{ others}}} \hfill \\ \end{array} } \right.{\text{ }}j = 1,2,3,4$$

In which, *CR* is crossover factor. The value of *CR* in the standard DE algorithm is fixed, typically taken as a constant between 0.5 and 1.0. A higher *CR* value improves the diversity ability of the algorithm, meanwhile reduces the ability of sensitive neighborhood search, and reduces the efficiency of search. A low *CR* value can facilitate sensitive neighborhood search, but it may reduce the diversity ability of the system and lead to the local optimal situation. This study underscores the importance of effectively adjusting the value of *CR.* The *CR* is dynamically adjusted during the search process as follows:

15 $$CR_{{i,g + 1}} = \left\{ {\begin{array}{*{20}l} {rand{\text{(0,1) }}f{\mathrm{(}}U_{{i,g}} {\text{) > }}f{\mathrm{(}}X_{{i,g}} {\mathrm{)}}} \hfill \\ {CR_{{i,g}} {\text{ }}f{\mathrm{(}}U_{{i,g}} {\mathrm{)}} \le f{\mathrm{(}}X_{{i,g}} {\mathrm{)}}} \hfill \\ \end{array} } \right.$$

(4) Selection:

Evaluate the objective function for the trial vector. If the trial vector is better than the current individual, replace the current individual with the trial vector; otherwise, keep the current individual.

16 $$X_{{i,g + 1}} = \left\{ {\begin{array}{*{20}l} {X_{{i,g}} {\text{ }}f{\mathrm{(}}U_{{i,g}} {\text{) > }}f{\mathrm{(}}X_{{i,g}} {\mathrm{)}}} \hfill \\ {U_{{i,g}} {\text{ }}f{\mathrm{(}}U_{{i,g}} {\mathrm{)}} \le f{\mathrm{(}}X_{{i,g}} {\mathrm{)}}} \hfill \\ \end{array} } \right.$$

The essence of response spectra calibration is to fit the design spectra according to ground motion spectra characteristics. Optimization problems should select appropriate optimization objectives to find the optimal parameters. According to the characteristics of response spectra calibration, the objective function for each candidate solution in the population is as follows:

17 $$f = \left\{ {\frac{1}{{T_{m} }}\int_{0}^{{T_{m} }} {[\beta (T) - \widetilde{\beta }(T)]^{2} } dT} \right\}^{{\frac{1}{2}}}$$

In which *f* is the root-mean-square error function, *T*_*m*_ represents the maximum spectral period.*β (T)* donates the records normalized response spectra value of a given spectral period *T*, and *β (T)* represents the calibrating spectra value of the same spectral period *T*.

**Fig. 4:**
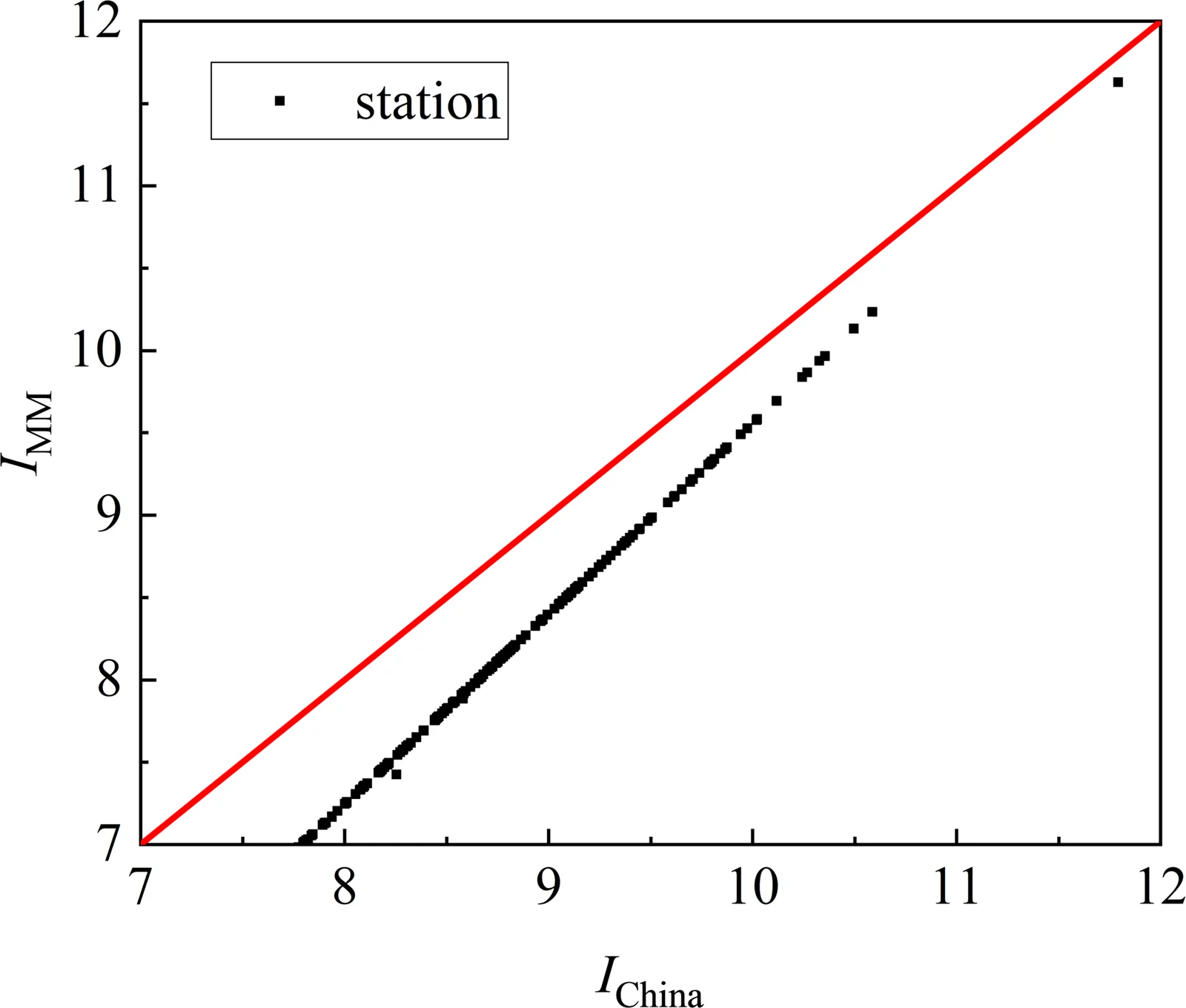
A comparison of Chinese instrumental seismic intensity (*I*_china_) and USGS instrumental seismic intensity(*I*_MM_).

Figure 4 presents the comparison between Chinese instrumental seismic intensity (*I*_**china**_) and USGS instrumental seismic intensity (*I*_*MM*_) of the stations selected in this study^33^. It can be observed that *I*_**china**_ and *I*_*MM*_ present a linear relationship. However, the Ichina is significantly higher than *I*_*MM*_, with *I*_**china**_ approximately one degree larger than *I*_*MM*_.

**Fig. 5:**
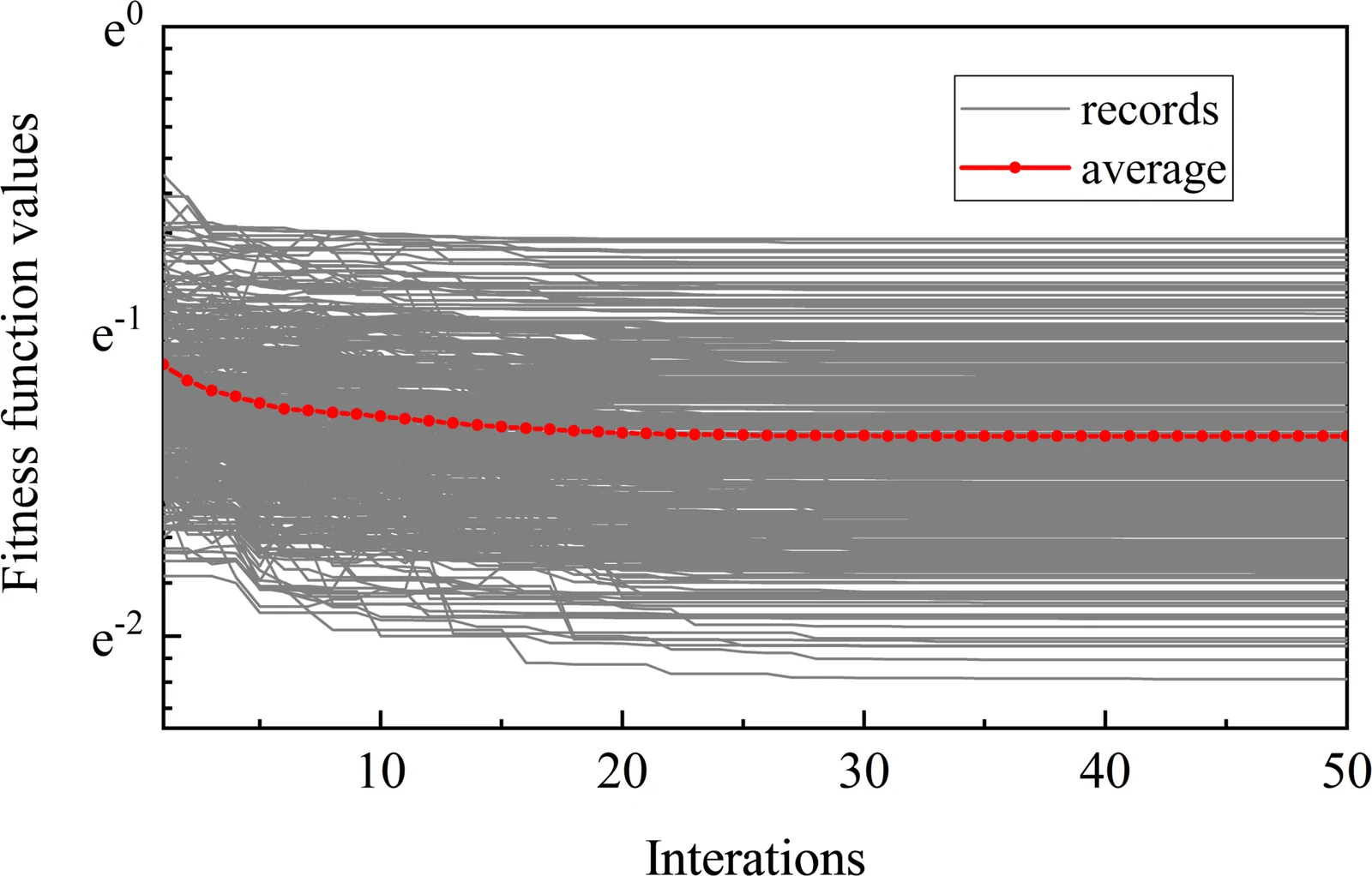
Trends of the objective function with respect to iterations.

Figure 5 illustrates the trend of the average objective function for all records and average values as the number of iterations increases. As depicted in Fig. 5, f initially decreases and then stabilizes with the increase in iterations. As the number of iterations increases, *f* for most records gradually decreases, falling within the ranges of e^-1^ to e^-2^. The first 10 iterations show a more obvious decline. When the number of iterations is between 30 and 50, *f* is stable in the range of e^-0^ to e^-2^, with a minimum value lower than e^-2^ and a maximum higher than e^-1^. The goodness of fit of the calibration design spectra can be improved by appropriately increasing the number of iterations. In this study, stable objective function values can be obtained by performing 50 iterations.

**Fig. 6:**
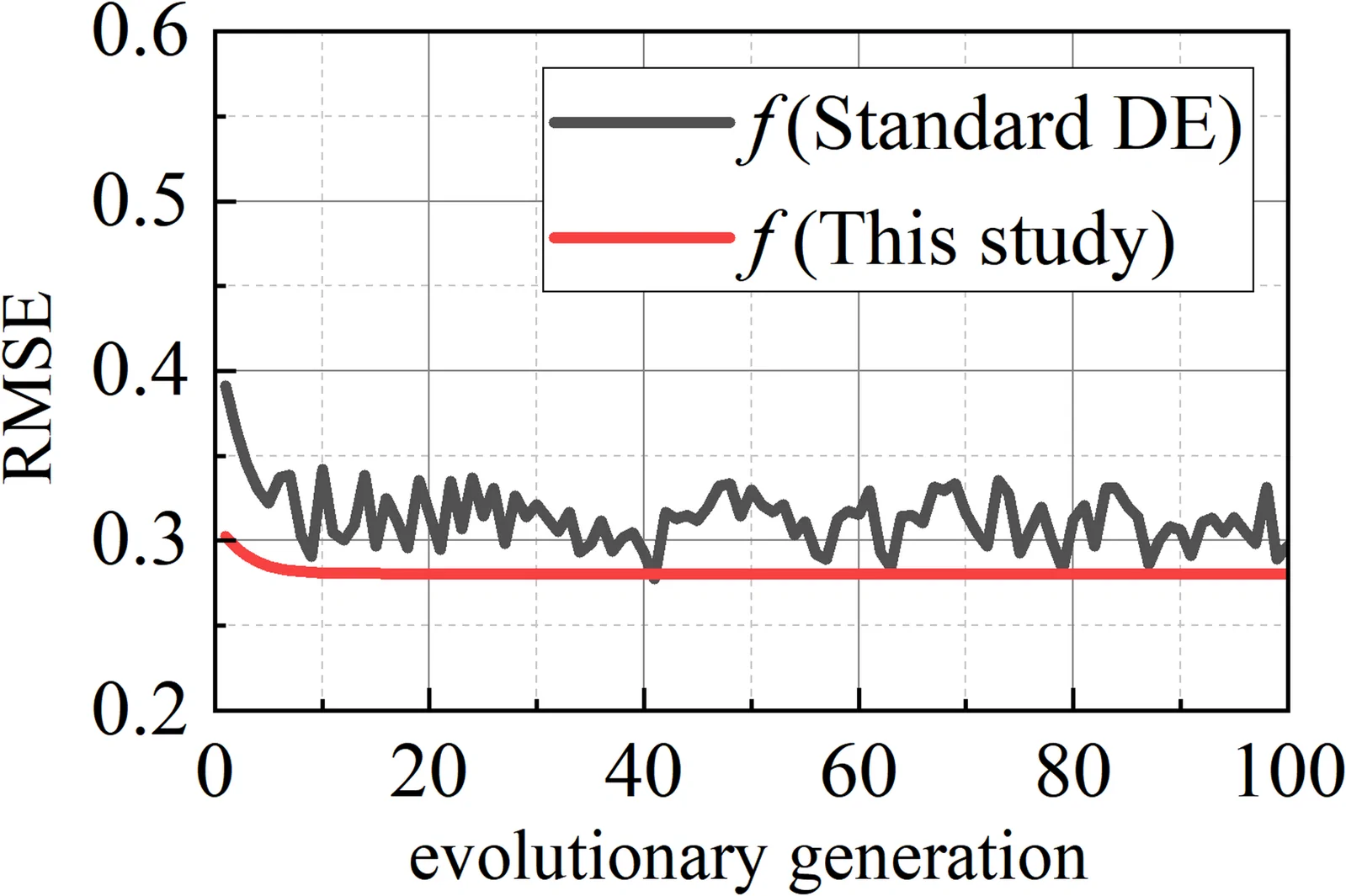
Convergence comparison of the proposed algorithm and the standard DE algorithm.

**Fig. 7:**
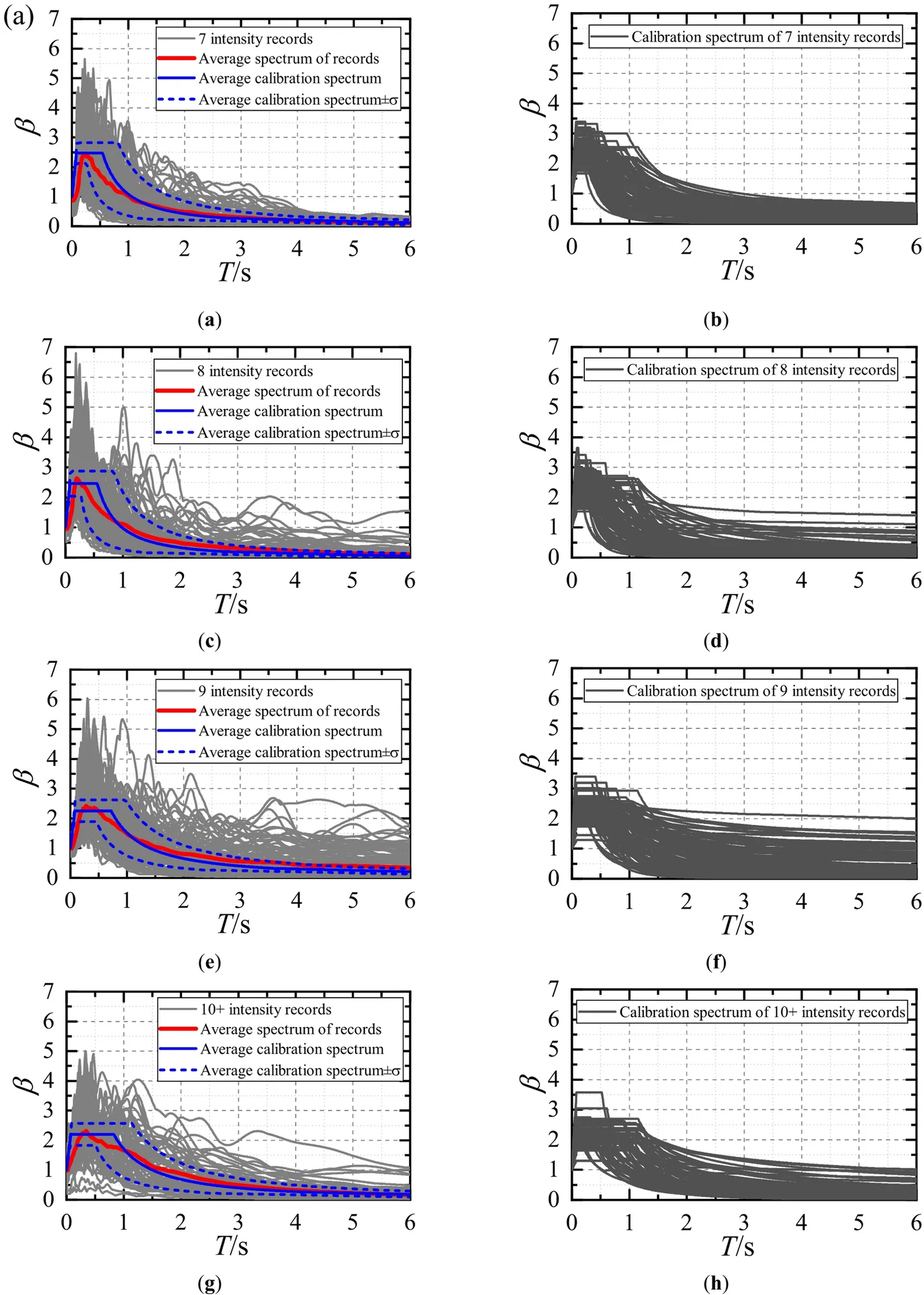
Calibration response spectrum of different intensities.

It is worth noting that the standard differential evolution (DE) algorithm suffers from premature convergence or stagnation problems, often falling into local optima. Its performance is also highly sensitive to the initial parameter settings, which are largely determined by human factors. To overcome these limitations, this study employs a hybrid mutation differential evolution algorithm with self‑adaptive control parameters for response spectrum calibration. This approach avoids the influence of manually set mutation and crossover parameters on the fitting results, thereby generating stable calculations and achieving fast convergence. Figure 6 compares the proposed algorithm with the standard DE algorithm in terms of the objective function (RMSE) over evolution generations. As can be seen from Fig. 6 and 7 the RMSE of the proposed algorithm decreases rapidly from generation 1 to 5 and remains stable after generation 50. In contrast, the RMSE of the standard DE algorithm declines rapidly in the first 20 generations, but then fluctuates and remains consistently larger than that of the proposed algorithm. These results demonstrate that the proposed algorithm outperforms the standard DE algorithm in both convergence speed and solution stability.

### Distribution of calibration parameters

It should provide a concise and precise description of the experimental results, their interpretation, as well as the experimental conclusions that can be drawn.

In order to in-depth investigate the relationship between calibration spectra parameters and the intensity, this study categorizes the calibration spectra parameters according to different intensities. Ground motion records of intensities ranging from 7 to 10 + were selected for analysis, and the trends of high intensity parameters are analyzed as follows.

**Fig. 8:**
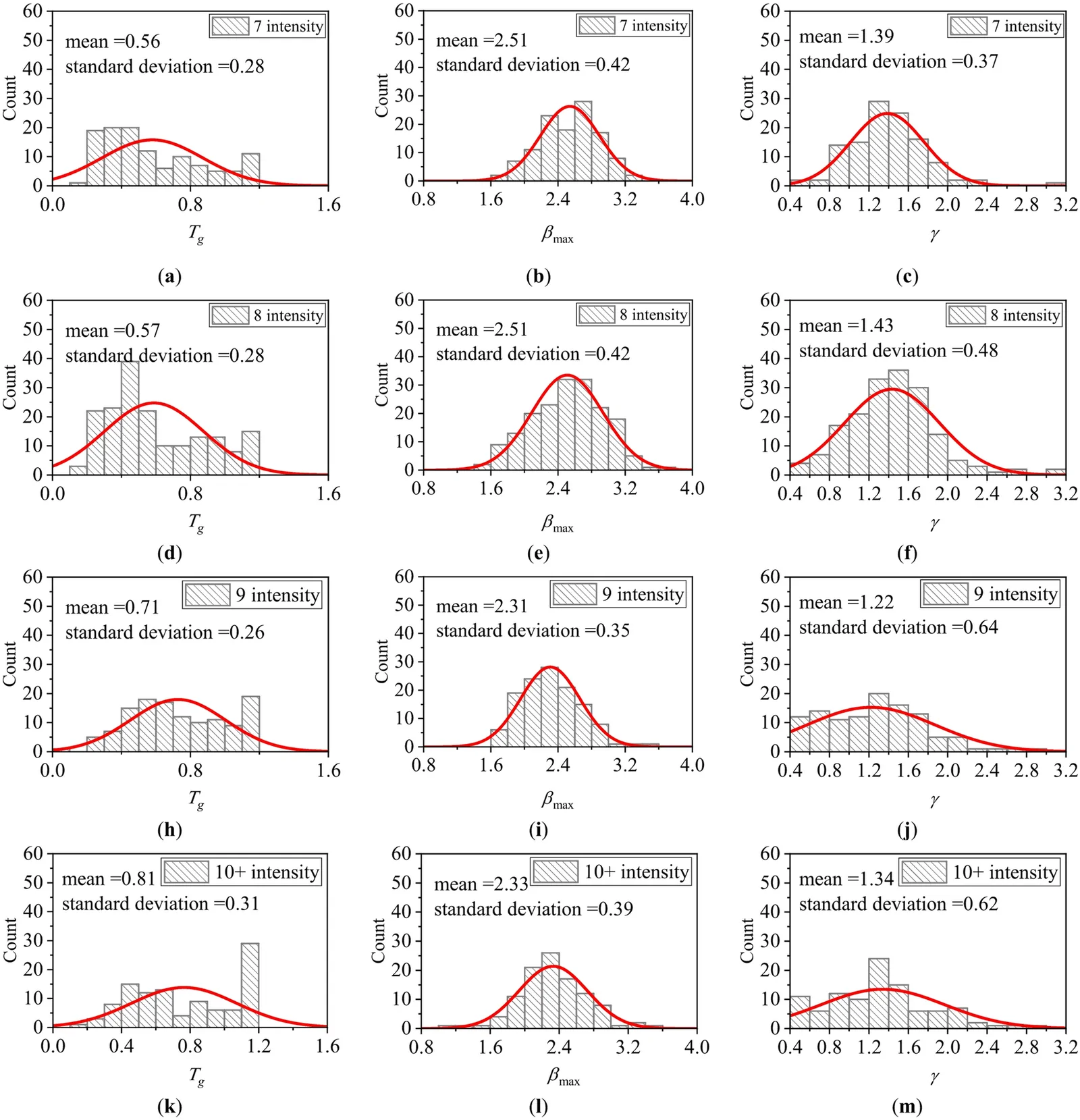
Distribution of calibration spectra parameters of different intensities.

Figure 8 shows the distribution of calibration spectra parameters for different intensities. The mean value and standard deviation of *T*_g_ for records with an intensity of 7 are 0.56 and 0.28, respectively, with *T*_g_ for most records falling between 0.2 and 1.2. The mean value and standard deviation of *T*_g_ for records with an intensity of 8 are similar to those of intensity 7. The mean value of *T*_g_ for records with an intensity of 9 is 0.71, which is significantly higher than that of intensity 7 and 8, while the standard deviation, which is 0.26, is similar. The mean value of *T*_g_ for records with an intensity of 10 + is approximately 0.81, which exceeds the mean value of *T*_g_ for intensities 7, 8, and 9, indicating that *T*_g_ significantly increases under the condition of high-intensity ground motions.

The mean value of *β*_max_ for records with an intensity of 7 is 2.54, the standard deviation is 0.35, and most records are in the range of 1.6–3.2. The mean value of *β*_max_ for records with an intensity of 8 is 2.51, the standard deviation is 0.42, and the *β*_max_ of most records is in the range of 1.6–3.2. The mean value of *β*_max_ for records with an intensity of 9 is 2.31, the standard deviation is 0.35, and *β*_max_ for most records is in the range of 1.6–3.2. The mean value of *β*_max_ for records with an intensity of 10 + is 2.33, the standard deviation is 0.39, and most records are in the range of 1.6-3.0. The mean value of *γ* for records with an intensity of 7 is 1.39, and the standard deviation is 0.37, with most values in the range 0.8-2.0. The mean value of *γ* for records with an intensity of 8 is 1.43, the standard deviation is 0.48, and most values are in the range of 0.8-2.0. The mean value of *γ* for records with an intensity of 10 + is 1.34, the standard deviation is 0.62, and most values are in the range of 0.8-2.0.

In general, the distributions of *β*_max_ for records with an intensity 7 and 8 are similar, as are the distributions of *β*_max_ for records with an intensity 9 and 10+. The *β*_max_ for intensity 7 is the largest among all four intensity groups, while the *β*_max_ for intensity 9 is the smallest. The average *β*_max_ for intensities 7 and 8 are greater than those for intensities 9 and 10+. The standard deviation of γ for intensities 9 and 10 + is greater than that for intensities 7 and 8. The distribution of *γ* is discrete. This indicates that the difference in the spectra values of the medium-long period (1–5 s) response spectra of different records increases significantly in strong ground motion.

### Influence of *V*_s30_ on calibration spectra parameters

Site conditions have an important impact on the design spectral parameters. According to the Code for Seismic Design of Buildings (GB50011-2010, hereafter CSDB2010), the characteristic period of the response spectra is in the range of 0.2–0.9 s according to different site classifications. This study studied the variation of the calibration spectra parameters with respect to *V*_s30_ and compared them with the response spectra parameters of the code (CSDB2010). It provides a reference for design spectra revision.

**Fig. 9:**
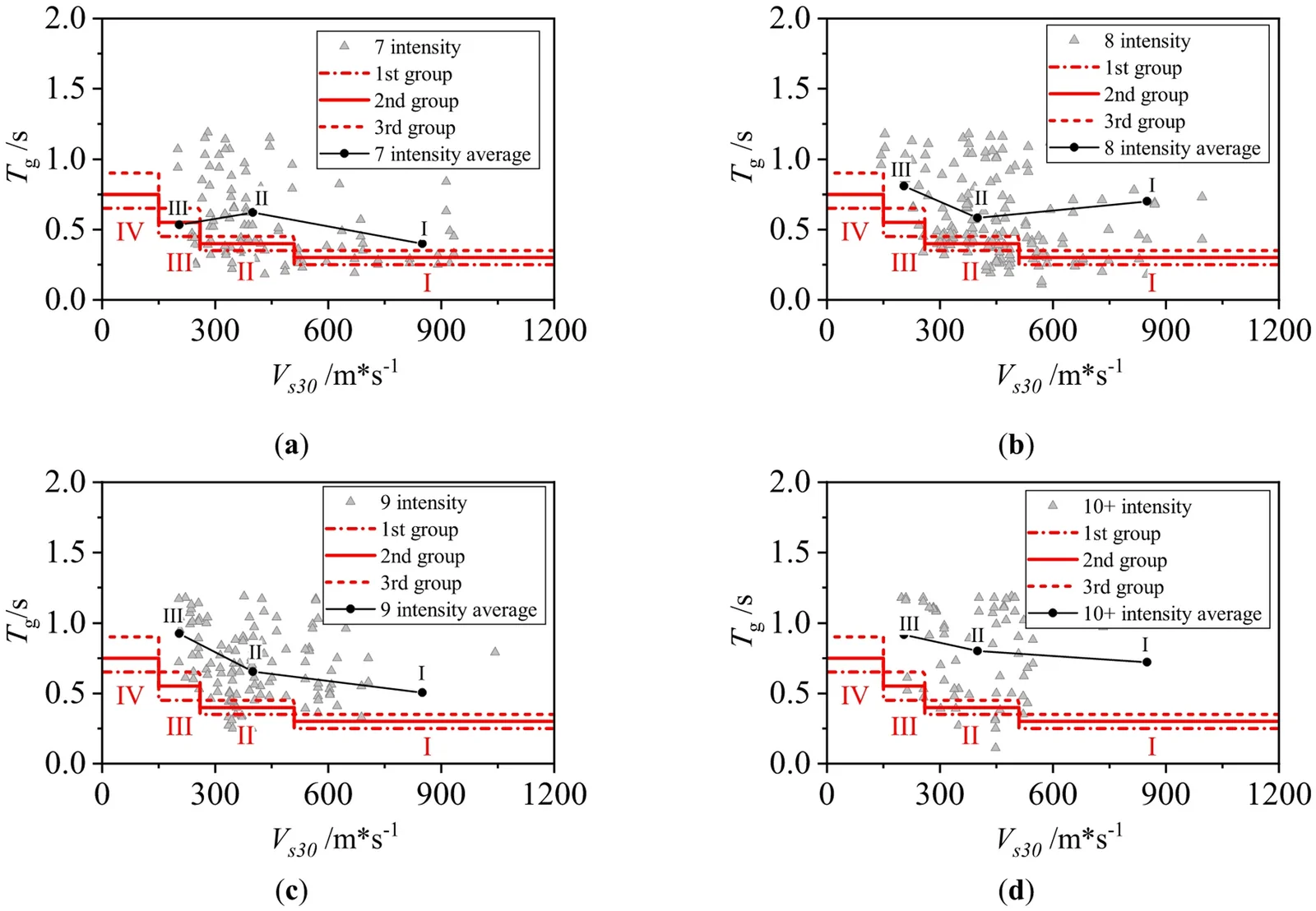
Distribution of site characteristic period (*T*_g_) of different intensities. The red lines are the site characteristic period (*T*_g_) of CSDB2010.

Figure 9 shows the *T*_g_ variation according to records with different intensities calibrated by this study. This study finds the trend of *T*_g_ with respect to *V*_s30_ and compares the *T*_g_ for records with the *T*_g_ of CSDB2010. The *T*_g_ of the CSDB2010 is related to the site’s condition classification and the seismic distance group. The majority of *T*_g_ for records in this study are significantly higher than *the T*_g_ of CSDB2010, while a few are near to or less than the *T*_g_ of CSDB2010. Correspondingly, the average *T*_g_ for records with intensity 7 is close to *T*_g_ of CSDB2010. The average *T*_g_ for records of intensity 8 is a little higher than the *T*_g_ of CSDB2010. Average *T*_g_ for records with intensity 9 and 10 + is obviously greater than the *T*_g_ of CSDB2010. For intensity 7, there are a few records that belong to the class III site. However, the average *T*_g_ of records of the III class site is similar to the *T*_g_ of CSDB2010. For the class II site, *T*_g_ for most records of intensity 7 is larger than the *T*_g_ of CSDB2010, and the average *T*_g_ is significantly higher than *T*_g_ of CSDB2010. For class I sites, the average *T*_g_ for records of intensity 7 is slightly higher than *T*_g_ of CSDB2010, and *T*_g_ for most records with intensity 7 are similar to *T*_g_ of CSDB2010. *T*_g_ for most records with intensity 7 is generally greater than the *T*_g_ of CSDB2010. while the *T*_g_ of a few records is similar to the *T*_g_ of CSDB2010. For different intensities, *T*_g_ of Class II and III sites are slightly larger than the *T*_g_ of CSDB2010, and *T*_g_ of Class I sites are obviously larger than *T*_g_ of CSDB2010. The *T*_g_ of CSDB2010 in Class I site and Class II site with different intensities site are small on the whole. Therefore, the influence of intensity on *T*_g_ should be considered, and the value of *T*_g_ should be increased in high intensity.

**Fig. 10:**
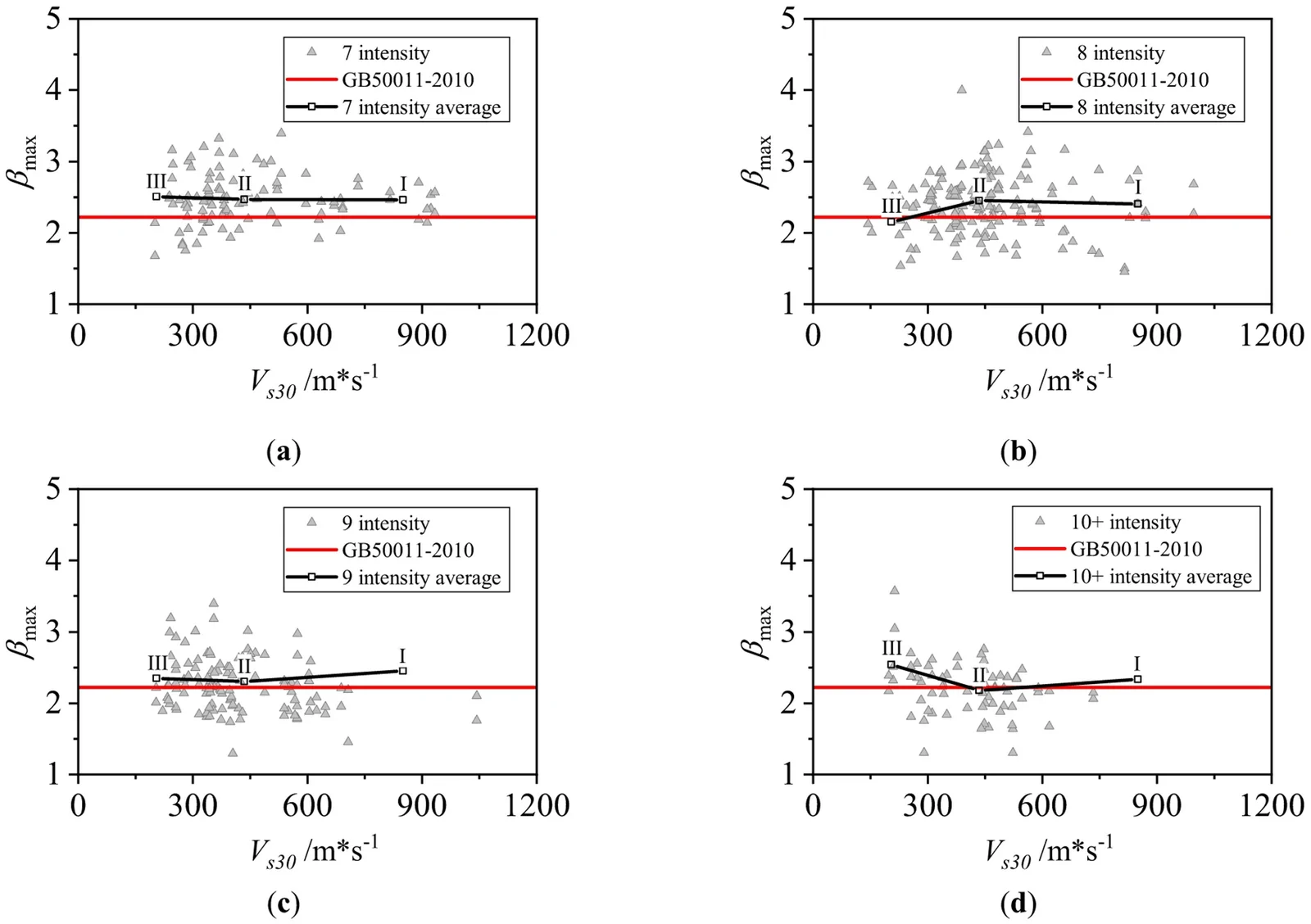
Distribution of the spectra platform value (βmax) of different intensities. The red lines are the spectra platform value (*β*max) of the code (CSDB2010). βmax (CSDB2010) = 2.25.

Figure 10 shows the *β*_max_ variation according to records with different intensities calibrated by this study. The average *β*_max_ of with different intensities is shown in the figure and compared with the *β*_max_ of CSDB2010. It can be seen from the figure that *β*_max_for most records are between 1.5 and 3, and *β*_max_ for records with different intensities are about 2.25. When in intensity 7, the average *β*_max_ of records calibration spectra in different sites was similar, both greater than the *β*_max_ of CSDB2010, and the average *β*_max_ of class III sites was the largest. When *V*_s30_ is less than 260 m/s, the average *β*_max_ for records with intensity 8 is less than 2.0, and the average *β*_max_ is more than the *β*_max_ of CSDB2010 when *V*_s30_ is more than 260 m/s. For records with intensity 9, the average value of *β*_max_ in class III and II sites is close to *β*_max_ of CSDB2010, which is similar to *β*_max_ of CSDB2010, while the average *β*_max_ in class I sites is significantly greater than *β*_max_ of CSDB2010. For records with intensity 10+, the average value of *β*_max_ in class III sites is significantly greater than 2.3, while that in class II and I sites is close to the *β*_max_ of CSDB2010. On the whole, with the intensity increasing, the average *β*_max_ in different class sites is gradually different from *β*_max_ of CSDB2010; records of class II site are the closest, and records of class I and III are significantly different from *β*_max_ of CSDB2010.

**Fig. 11:**
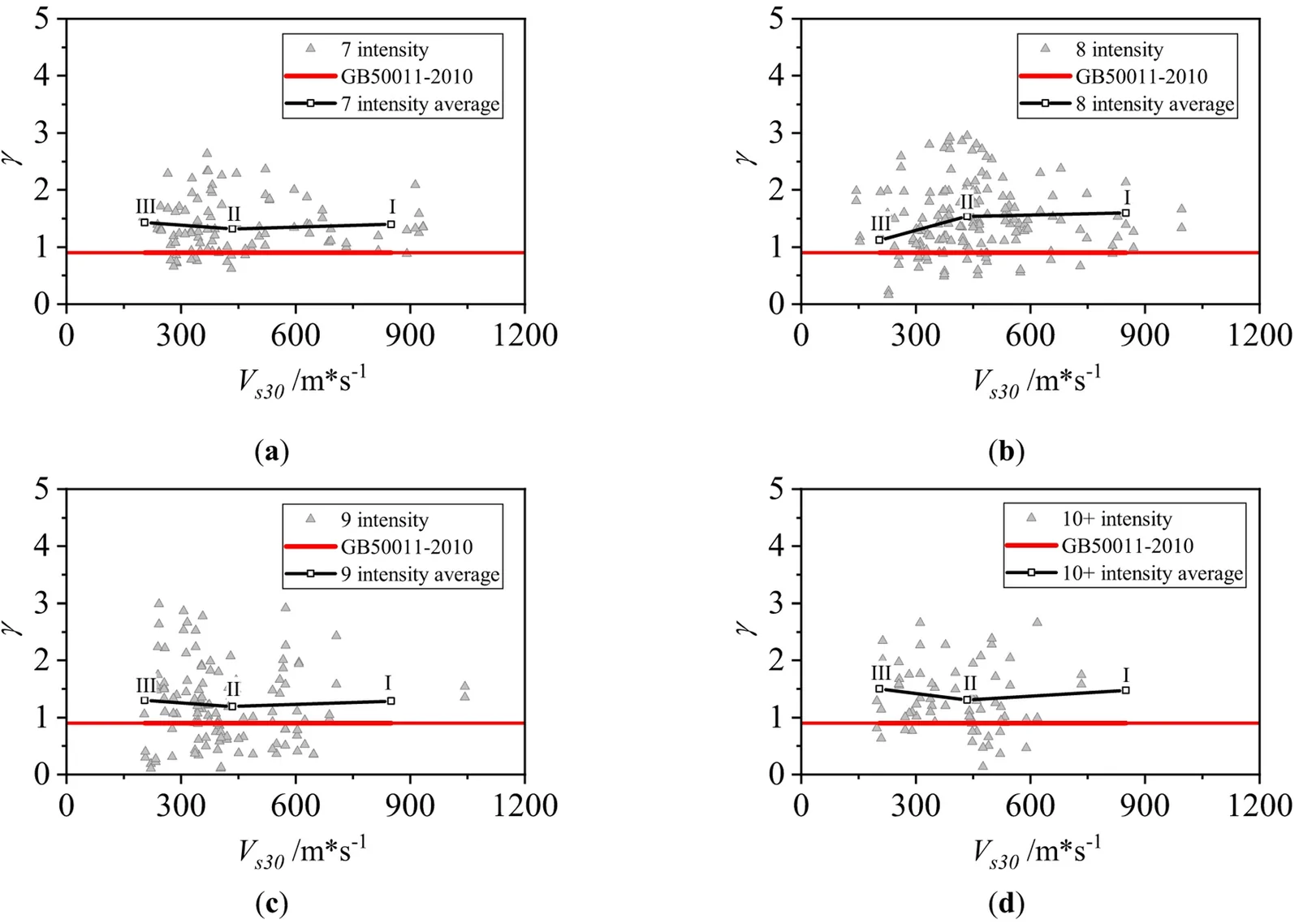
Distribution of the attenuation index (*γ*) of different intensities. The red lines are the attenuation index (*γ*) of code (CSDB2010). *γ* (CSDB2010) = 0.9.

Figure 11 shows the distribution of *γ* with respect to *V*_s30_. The average *γ* for records with different intensities is shown in the figure and compared with the *γ* of CSDB2010. It can be seen from the figure that most of the calibration records spectra *γ* are between 0.7 and 2.5. For records of 7 intensity, most of the *γ* are greater than the *γ* of CSDB2010, and the average *γ* of different sites is 1.2. For records of intensity 8, the average *γ* in Class III sites is the closest to *γ* of CSDB2010, while that in Class II and I sites is significantly higher than 0.9. When the intensity is 9, the distribution of *γ* is relatively discrete, and the *γ* of some records is less than 0.9, while the average value of *γ* in different sites is close to 0.9. When the intensity is 10+, the average *γ* of all sites is greater than the *γ* of CSDB2010. On the whole, the average value of *γ* with different intensities in this study is greater than *γ* of CSDB2010.

### Compare with the ground motion prediction equation (GMPE)

The ground motion prediction equation is the basis of seismic ground motion parameter zonation, including source parameter, path parameter, and site parameter, which can predict ground motion parameters for potential earthquakes in a region. GMPE is of great significance for the seismic design of a region. In this study, the calibrated response spectra are compared with those of GMPE^37–40^.

**Fig. 12:**
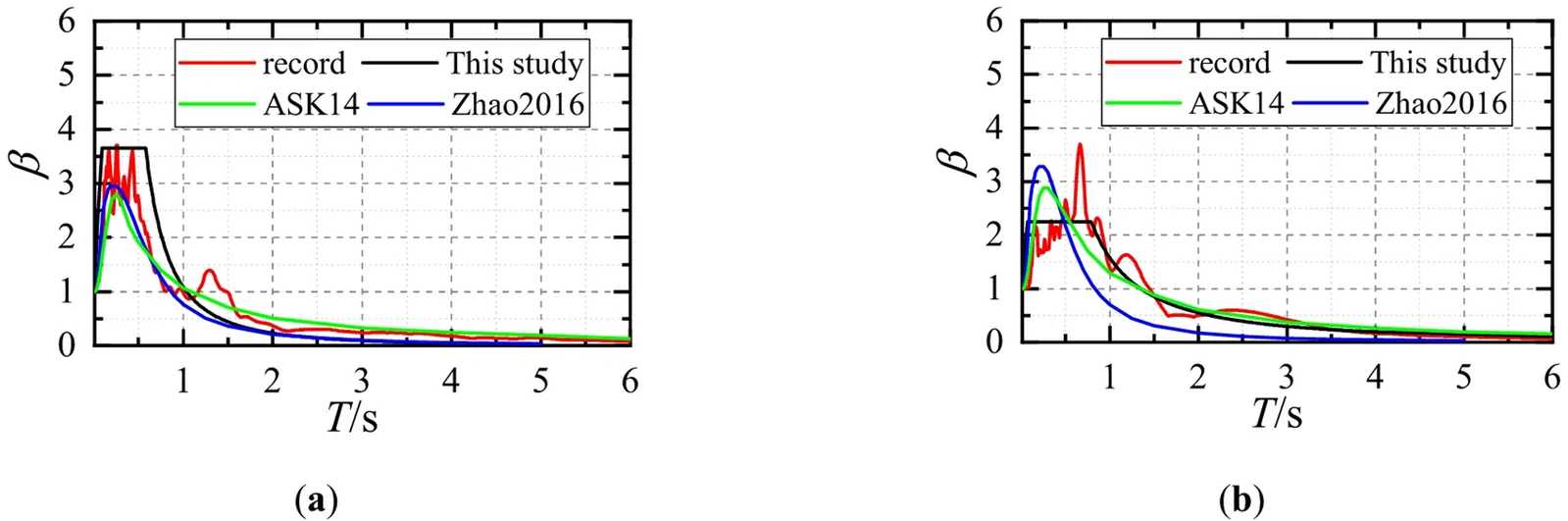
Comparison of calibrated response spectra with GMPEs: (**a**) Chi-Chi earthquake record (Mw 7.6, distance 15 km, *V*_s30_ 471 m/s, intensity 8); (**b**) Kobe earthquake record (Mw 6.9, distance 11 km, *V*_s30_ 256 m/s, intensity 9).

Two typical records were selected to be compared with the calibrated response spectra in this study and two GMPEs. In the perspective of the record of the Chi-Chi earthquake, as Fig. 12 (a) showing, In the spectral period range of 0.01–0.1 s, the value from this study is higher than those of the response spectra, while the two GMPEs are close to the record response spectra. In the spectral period range of 0.1–0.5 s, the value of the response spectra of this study is higher than the peak value of the response spectra, and the response spectra of Abrahamson et al.^37^. (hereafter ASK14) and Zhao et al.^38–40^(hereafter Zhao2016) are lower than the peak value of the response spectra. All the spectra fit well with the record response spectra. The values from ASK14 and Zhao2016 are significantly higher than the record response spectra in the medium and short period, and are closer to the record response spectra in the long period. However, values of ASK14 and Zhao2016 are slightly lower than the record response spectra in the medium and long spectra period. Another comparison for the typical record of the Kobe earthquake is presented in Fig. 12 (b). In the spectra period of 0–0.1 s, both the studied and GMPEs values are higher than the actual response spectra. The value of Zhao2016 is the largest in the spectral period of 0.1–0.6 s, and the value ofASK14 is the smallest in this study. When the spectra period exceeds 1.0s, the values of this study are similar to those of record spectra, while the values of ASK14 and Zhao2016 are smaller than those of record response spectra. Compared with GMPE, the response spectra calibrated in this study is more consistent with the trend of the record spectra. The characteristic periods (Tg) calculated in this study are generally higher than the standard values specified in current seismic design codes, and this discrepancy is significantly correlated with the dataset filtering criteria adopted in this work. To focus on the attenuation characteristics of high-intensity ground motions, this study only includes ground motion records from earthquakes with magnitude Mw > 6.5, and does not cover small-magnitude earthquake events that may also produce high-intensity ground motions in their near-field regions.

Mechanistic explanation for this discrepancy can be derived from existing GMPE studies: under the same observation distance, the source rupture width of large earthquakes is significantly larger than that of small earthquakes. A larger source rupture scale can excite more abundant long-period ground motion components, which consequently prolongs the characteristic period of ground motions. This mechanism indicates that direct application of Tg values calculated based on moderate and strong earthquake records to seismic design of engineering structures in the near-field area of small earthquakes may cause a certain degree of overestimation of the actual characteristic period.

The Tg value conclusions of this study are more applicable to seismic design checking of engineering structures in areas affected by moderate and strong earthquakes. Future studies can further incorporate high-intensity near-field records from small-magnitude earthquakes to improve the characteristic period value system for different magnitude intervals, and provide more accurate parameter basis for seismic design of engineering structures under different magnitudes and distance conditions.

## Conclusions

In this study, the Differential Evolution (DE) algorithm is used to calibrate the near-fault records of strong ground motions. Three objective functions (root-mean-square error function) are selected and compared with the objective function of the Differential Evolution (DE) algorithm, and the relationship between calibration response spectrum parameters and site *V*_s30_ was analyzed. Conclusions were drawn as follows:

1. The calibrated design spectrum in this study is based on ground motion records. Compared with GMPE, the calibrated response spectrum in this study is more consistent with the trend of the record response spectrum.
2. Average *T*_g_ values of Class I and Class II sites are greater than the *T*_g_ of the code (CSDB2010). This study suggests that the influence of seismic intensity on *T*_g_ should be considered, and *T*_g_ should be increased for 8, 9, and 10 + intensities. The average *T*_g_ of 10 + intensity is greater than the average *T*_g_ of 7, 8, and 9 intensities, indicating that the site characteristic period will increase with the increase of intensity.
3. Most *β*_max_ values of records range from 1.5 to 3, and *β*_max_ of different intensities is similar to 2.25, among which class II is the closest, class I and class III sites are significantly different from the *β*_max_ of CSDB2010. when the intensity is 7 degrees, the calibrated *β*_max_ value of the record is significantly higher than the *β*_max_ of CSDB2010. The average *β*_max_ of 7 intensity records and 8 intensity records is close to each other. The average *β*_max_ of 9 intensity records and 10 + intensity records is close to each other. The average *β*_max_ of 7 intensity records is the largest, and the average *β*_max_ of 7 intensity records is the smallest.
4. Most of the calibration records spectra *γ* are between 0.7 and 2.5. The average *γ* of 7, 8 intensity records are greater than that of intensity 9 and intensity 10, and the standard deviation of *γ* of intensity 9 and intensity 10 is greater than that of intensity 7 and intensity 8, indicating that *γ* is more discrete in the case of high intensity.

## Data Availability

We use data from the Center for Engineering Strong Motion Data (CESMD), providing the Engineering Strong Motion Data from the network publicly available at https: //www.strongmotioncenter.org. We use data from the Pacific Earthquake Engineering Research Center (PEER), publicly available at https://ngawest2.berkeley.edu/site. Data from the Disaster and Emergency Presidency of Turkey (AFAD) for providing the Engineering Strong Motion Data from the network is publicly available at https://tadas.afad.gov.tr/login. Data from the National Research Institute for Earth Science and Disaster Resilience (NIED) is publicly available at [https://www.kyoshin.bosai.go.jp](https:/www.kyoshin.bosai.go.jp) .
